# Socioeconomic Position and Oral Health in Chinese Older Adults: A Life Course Approach

**DOI:** 10.1177/23800844241297533

**Published:** 2024-12-09

**Authors:** J. Hong, R.G. Watt, G. Tsakos, A. Heilmann

**Affiliations:** 1 Department of Epidemiology and Public Health, University College London, London, UK; 2 National Institute for Health Research Applied Research Collaboration West, University Hospitals Bristol and Weston NHS Foundation Trust, Bristol, UK; 3 Population Health Sciences, Bristol Medical School, University of Bristol, Bristol, UK

**Keywords:** tooth loss, geriatric dentistry, social inequalities, epidemiology, structural equation modelling, life course perspective

## Abstract

**Objectives:**

We investigated associations between socioeconomic position (SEP) across childhood, adulthood, and older age and number of teeth among Chinese older adults.

**Methods:**

Data came from 15,136 participants aged 65 to 105 y in the Chinese Longitudinal Healthy Longevity Survey (2018 wave). The outcome was number of teeth. Pathways and sensitive period models were tested simultaneously via structural equation modeling. Ordinal logistic regression assessed the accumulation of risk and social mobility models. Differences were examined across 4 birth cohorts.

**Results:**

Adult and older age SEP had direct effects on number of teeth in older age (adulthood, direct β = 0.182, *P* < 0.001; older age, direct β = 0.093, *P* = 0.005), supporting the sensitive period model. Childhood SEP had an indirect effect on number of teeth (indirect β = 0.130, *P* < 0.001) through adult and older age SEP, supporting the pathway/accumulation of risk and social mobility models. Effects of SEP on number of teeth were more pronounced in younger cohorts. Graded associations in the expected directions were found between the number of periods in which participants experienced disadvantaged SEP and number of teeth, as well as social mobility trajectories and number of teeth.

**Conclusion:**

Among Chinese older adults, the number of remaining teeth is subject to marked social inequalities. Our findings document the simultaneous applicability of life course models and a widening of oral health inequalities in China across generations. Interventions earlier in child and adult life are needed to address this problem and reduce oral health inequalities.

**Knowledge Transfer Statement:**

The findings of this study suggest marked socioeconomic inequalities in oral health among Chinese older adults. These inequalities are generated throughout the life course and appear to have widened across cohorts. This study emphasizes that interventions are needed to address the social determinants of oral health at all life stages.

## Introduction

Good oral health is an important part of healthy aging. Oral conditions are among the most prevalent causes of disability-adjusted life-years among older populations globally (Vos et al. 2020). Among adults aged 65 to 74 y in China, the prevalence of edentulousness decreased from 6.8% in 2005 to 4.5% in 2015, but the prevalence of good periodontal status, defined according to the 2018 classification of periodontal disease, decreased from 14.1% to 9.3% (Chinese Stomatological Association 2018). The increasing burden of oral diseases and other chronic conditions, coupled with China’s fast-aging population, will put further strain on the nation’s resources, while oral health inequalities are considerable and increasing (Zhang and Chen 2019).

A life course perspective to oral conditions is appropriate, given that they are chronic, highly prevalent, and cumulative and can be reliably measured (Nicolau et al. 2007; Heilmann et al. 2015). Life course conceptual models explain how exposures across the life span influence later-life morbidity (Ben-Shlomo and Kuh 2002). The sensitive period model applies to exposures that have an effect or stronger effect on a disease outcome during a certain time window, irrespective of circumstances in other periods. The accumulation of risk model suggests that adverse exposures are accumulated incrementally over the life span. The pathway or chain-of-risk model indicates that earlier risk factors influence subsequent health outcomes indirectly via later risk factors. An alternative approach for conceptualizing accumulation of risk, the social mobility model, considers how the lifetime movement of an individual within social hierarchies may affect later-life health (Hallqvist et al. 2004).

Oral health life course studies have mostly employed traditional regression analyses (Pearce et al. 2004; Bernabé et al. 2011; Holst and Schuller 2000; Gulcan et al. 2015; Green and Popham 2017; Han and Khang 2017; Ramsay et al. 2018; Lee 2019; Zhang and Chen 2019), which are methodologically limited and may underestimate associations (Moosbrugger et al. 2009; De Stavola et al. 2015). Application of more advanced techniques, such as theory-driven structural equation modeling (SEM), can elucidate the pathways linking life course socioeconomic position (SEP) and oral health while accounting for measurement error and intermediate confounders (Moosbrugger et al. 2009; Baker and Gibson 2014; De Stavola et al. 2015). Only 2 studies have used SEM to decompose the direct and indirect effects of lifetime SEP on tooth loss in later life, suggesting that childhood SEP affects later-life tooth loss mainly indirectly via adult SEP (Celeste et al. 2020; Zhang et al. 2023).

Existing evidence on associations between life course SEP and oral health comes mainly from high-income countries (HICs), while the topic is underinvestigated in low- and middle-income countries, including China (Zhang and Chen 2019). Social stratification and social mobility in China differ considerably from those in HICs, partly because China’s political and economic system underwent extensive historical changes (Bian 2002). Testing the applicability of life course theories to populations in diverse cultural and socioeconomic settings could enrich the broader understanding of health inequalities and inform interventions that can be adapted to various contexts. Another underresearched area is the existence of cohort effects (generational differences) in the association between SEP and later-life oral health in China, which underwent significant historical changes that were experienced differently across birth cohorts (Ahacic et al. 2007). A recent study examined pathways from childhood SEP to edentulousness among adults in the China Health and Retirement Longitudinal Study but did not include old age SEP or investigate cohort effects (Zhang et al. 2023). Moreover, no research has examined oral health inequalities among people aged ≥80 y in China. Given the global trend of an aging population, significant gaps in the evidence exist that require addressing. The current study aimed to test the applicability of different life course models to number of teeth in a nationally representative sample of older Chinese aged 65 to 105 y. We also examined whether associations varied among 4 birth cohorts. We hypothesized that childhood and adult SEP would affect the number of teeth directly and indirectly via older age SEP and that those who experienced more socioeconomic disadvantage over the life course would have fewer teeth in old age. In addition, we anticipated cohort effects, with number of teeth being more socially patterned among younger cohorts.

## Materials and Methods

### Study Population

We used cross-sectional data from the latest wave (2018) of the Chinese Longitudinal Healthy Longevity Survey (CLHLS; Center for Healthy Aging and Development Studies 2020). Participants aged ≥65 y were randomly selected from 631 counties and cities in 23 of 31 provinces, representing approximately 85% of the Chinese population (Zeng et al. 2008). The CLHLS adopted a multistage disproportionate and targeted random sampling method (Appendix 1). Centenarians and nonagenarians were oversampled. Those older than 105 y were excluded due to a lack of sampling weights. Data on sociodemographic characteristics, life satisfaction and personality, cognitive ability, health behaviors, ability to perform daily activities, and physical health were collected through face-to-face interviews and physical examinations from 2018 to 2019. Further details on sampling design and data quality can be found elsewhere (Zeng et al. 2008).

### Socioeconomic Position

SEP, the exposure, was measured at 3 life stages: childhood, adulthood, and older age. Childhood SEP was assessed retrospectively via father’s education (0, ≥1 y) and main occupation (manual or unemployed, nonmanual). Adult SEP was retrospectively measured through participants’ education (0, 1 to 6, ≥7 y) and main occupation before the age of 60 y (manual or unemployed, nonmanual). Older age SEP was composed of self-rated economic status at the time of the survey and annual household income in the previous year (2017). Self-rated economic status was based on the question “How would you rate your economic status compared with neighbours?” Answers were categorized as poor or very poor, fair, and good or very good. The CLHLS did not code the exact value of income >100,000 Chinese yuan. Therefore, annual household income tertiles were used: low, <10,000 (equivalent to <1,476 US dollars); medium, 10,000 to 50,000 (1,476 to 7,396); and high, >50,000 (>7,396).^1^

Cumulative disadvantage was measured by summing the number of periods in which participants experienced disadvantaged SEP,^2^ resulting in 4 categories: disadvantaged SEP in 0, 1, 2, and 3 periods. Based on this, a social mobility variable was created with 5 possible trajectories: stable advantaged (high-high-high), upward (low-high-high or low-low-high), fluctuating (low-high-low or high-low-high), downward (high-high-low or high-low-low), and stable disadvantaged (low-low-low).

### Number of Teeth in Older Age: Outcome

Tooth loss reflects the accumulation of oral disease over the life course. Previous validation studies of oral health measures supported the validity of using self-reports to measure the number of natural teeth (Shen et al. 2013; Matsui et al. 2017). Participants were asked to count the number of natural teeth present (including fixed prosthetics) at the time of the 2018 survey, assisted by medical personnel (Center for Healthy Aging and Development Studies 2020). Number of teeth ranged from 0 to 32 and was categorized into 4 ordered groups for all analyses: 0 teeth (edentulousness), 1 to 9, 10 to 19, and 20 to 32 (functional dentition) (Appendix 2).

### Covariates

Covariates included age, sex, urban/rural residence, and region. Regions were categorized according to the National Bureau of Statistics of China: east, central, and west China. Urban/rural residence and region were included because of their considerable variation in economic development and health care accessibility.

### Statistical Analysis

The percentage of incomplete data was 16%. Multiple imputation of missing data on all exposures and covariates was carried out by the chained equation approach in Stata/SE version 17 (StataCorp). Appendix 3 presents the diagnostic plots of the distribution of the SEP variables for all 35 imputations, showing that the values vary randomly. The results obtained from complete case analyses and analyses with multiple imputation were similar (Appendix 3).

Chi-square tests estimated the crude associations between each independent variable and number of teeth (as summarized with proportion estimate and SE). Sampling weights were used to account for the survey design and ensure comparability of the sample with the standard population on age, rural/urban residence, and sex distribution. Multicollinearity diagnostics indicated noncollinearity between the SEP measures (Appendix 3).

**Figure 1.**
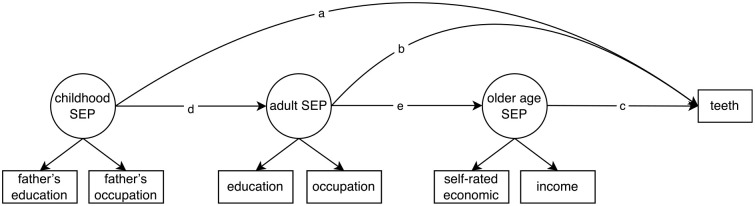
Analytic model for structural equation modeling analysis on the association between socioeconomic position (SEP) and number of teeth.

To test the 4 socioeconomic life course theoretical models, SEM was carried out with Mplus version 8.4. SEM can work with observed and latent variables, model direct and indirect effects, and take measurement errors into account (Weston and Gore 2006). Figure 1 shows the analytic model. Childhood SEP, a latent variable evaluated by fathers’ education and occupation, was modeled as having a direct effect on number of teeth through path *a* and indirect effects through paths *dec* and *db*. Adult SEP, measured by participants’ education and main occupation, was modeled to have a direct effect (path *b*) and indirect effect (path *ec*). Older age SEP, consisting of self-rated economic status and annual household income, was modeled as having only a direct effect through path *c*. Multiple-group analysis was performed to examine whether associations varied among the 4 birth cohorts. The following indices were considered indicators of good model fit: root mean square error of approximation ≤0.06, standardized root mean squared residual ≤0.08, and comparative fit index and Tucker-Lewis index ≥0.95 (Hu and Bentler 1999). Weighted least squares mean and variance estimation was used to fit categorical data, and 35 imputed data sets were imported from Stata/SE version 16 to address this method’s limitations of handling missing data. Theta parameterization was used, as recommended when estimating residuals for polytomous outcomes and multiple-group analysis (Muthén and Asparouhov 2002). Bootstrap assessment with 500 repetitions was conducted to assess estimation uncertainty. All models adjusted for demographic characteristics.

To explore the social mobility model, ordinal logistic regression was performed with partial proportional odds models. First, the associations between social mobility trajectories and number of teeth were examined, adjusting for demographic characteristics. Second, subgroup analyses stratified by birth cohort were conducted. Odds ratios (ORs) were reported with their 95% CIs. Accumulative risk models could also be tested by traditional regression models. We conducted a sensitivity analysis estimating a fully adjusted regression model, with cumulative disadvantage as the exposure.

## Results

Table 1 presents the distribution of number of teeth by SEP and demographic characteristics of 15,136 participants aged 65 to 105 y. Overall, 15.3% of participants were edentulous, 18.7% had 1 to 9 teeth, 19.7% had 10 to 19 teeth, and 46.3% reported having functional dentition (≥20 teeth). Women, older cohorts, those living in rural areas, and those from the western region of China had a higher prevalence of edentulousness and lower prevalence of functional dentition. These findings were socially patterned, with edentulousness most common and functional dentition least common among participants with lower SEP and those who experienced more periods of socioeconomic disadvantage in their lives.

**Table 1.** Descriptive Statistics for People Aged 65 to 105 y in 2018 by Number of Teeth (*n* = 15,136).

			No. of Teeth, Row % (SE)^a^				
	Unweighted *n*	Column % (SE)^a^	0	1 to 9	10 to 19	20 to 32	*P* Value
**Overall**			15.3 (0.4)	18.7 (0.5)	19.7 (0.5)	46.3 (0.7)	
**Demographic characteristics**							
Sex							<0.001
Male	6,659	47.7 (0.7)	13.8 (0.6)	17.3 (0.7)	19.9 (0.7)	49.0 (0.9)	
Female	8,477	52.3 (0.7)	16.8 (0.6)	19.9 (0.7)	19.4 (0.7)	43.9 (0.9)	
Birth year							<0.001
1941 or later	4,289	74.1 (0.4)	10.7 (0.5)	14.9 (0.6)	19.8 (0.7)	54.6 (0.8)	
1931 to 1940	4,069	21.7 (0.4)	25.9 (0.7)	28.5 (0.7)	20.5 (0.7)	25.1 (0.7)	
1921 to 1930	3,779	4.1 (0.1)	41.4 (1.0)	33.4 (1.0)	14.1 (0.8)	11.2 (0.7)	
1920 or earlier	2,999	0.2 (0.0)	57.3 (1.8)	30.1 (1.7)	7.0 (0.9)	5.6 (0.9)	
Residential place							<0.001
Rural	6,721	49.5 (0.7)	17.0 (0.6)	19.0 (0.7)	19.9 (0.8)	44.1 (1.0)	
Urban	8,415	50.5 (0.7)	13.7 (0.5)	18.3 (0.6)	19.5 (0.7)	48.5 (0.9)	
Region							<0.001
Eastern	7,569	50.0 (0.6)	16.1 (0.6)	18.0 (0.6)	20.7 (0.7)	45.1 (0.9)	
Central	3,851	26.1 (0.6)	12.8 (0.7)	18.3 (0.9)	17.3 (1.0)	51.5 (1.3)	
Western	3,716	23.8 (0.6)	16.4 (0.9)	20.3 (1.0)	20.1 (1.1)	43.1 (1.4)	
**Childhood SEP**							
Father’s education, y							<0.001
0	11,611	75.4 (0.6)	16.6 (0.5)	19.4 (0.5)	19.6 (0.6)	44.5 (0.8)	
≥1	2,617	24.6 (0.6)	11.5 (0.7)	16.5 (0.9)	20.0 (1.0)	52.0 (1.3)	
Father’s occupation in childhood							0.049
Manual or unemployed	14,237	95.0 (0.3)	15.6 (0.4)	18.6 (0.5)	19.7 (0.5)	46.0 (0.7)	
Nonmanual	567	5.0 (0.3)	10.2 (1.5)	19.3 (2.3)	18.5 (2.3)	52.0 (3.0)	
**Adult SEP**							
Own education, y							<0.001
0	7,568	30.3 (0.6)	21.0 (0.8)	25.0 (0.9)	18.9 (0.9)	35.1 (1.1)	
1 to 6	4,875	41.5 (0.7)	15.7 (0.7)	18.6 (0.8)	20.0 (0.8)	45.8 (1.0)	
≥7	2,705	28.2 (0.6)	8.7 (0.6)	12.0 (0.8)	20.1 (1.0)	59.2 (1.2)	
Main occupation							<0.001
Manual or unemployed	13,597	89.1 (0.4)	16.2 (0.4)	19.3 (0.5)	19.7 (0.5)	44.8 (0.7)	
Nonmanual	1,542	10.9 (0.4)	8.5 (0.9)	13.2 (1.2)	19.6 (1.5)	58.7 (1.8)	
**Older age SEP**							
Annual household income, ¥							0.001
Low, <10,000	4,632	34.1 (0.6)	18.1 (0.8)	20.8 (0.8)	19.6 (0.9)	41.5 (1.1)	
Medium, 10,000 to 49,999	4,709	34.7 (0.7)	15.7 (0.7)	19.0 (0.8)	19.7 (0.9)	45.6 (1.2)	
High, ≥50,000	4,484	31.1 (0.6)	11.9 (0.7)	15.9 (0.8)	19.8 (0.9)	52.4 (1.2)	
Self-rated economic status							<0.001
Poor	1,614	10.5 (0.4)	18.4 (1.4)	21.1 (1.5)	22.4 (1.7)	38.1 (2.0)	
Fair	10,440	70.9 (0.6)	15.2 (0.5)	19.3 (0.6)	19.4 (0.6)	46.1 (0.8)	
Good	2,912	18.6 (0.5)	14.3 (0.9)	14.7 (0.9)	19.1 (1.1)	51.8 (1.5)	
**Disadvantaged SEP in**							<0.001
0 periods	1,996	22.0 (0.6)	10.2 (0.7)	14.9 (1.0)	20.3 (1.1)	54.6 (1.4)	
1 period	4,624	47.4 (0.7)	14.5 (0.6)	16.7 (0.7)	19.4 (0.8)	49.4 (1.0)	
2 periods	5,413	28.1 (0.6)	20.0 (0.8)	24.3 (0.9)	19.5 (0.9)	36.2 (1.2)	
3 periods	728	2.6 (0.2)	24.6 (3.0)	24.8 (2.9)	21.2 (3.3)	29.4 (3.9)	
**Social mobility**							<0.001
Stable advantage	1,996	22.0 (0.6)	10.2 (0.7)	14.9 (1.0)	20.3 (1.1)	54.6 (1.4)	
Upward	9,143	69.0 (0.6)	16.3 (0.5)	19.2 (0.6)	19.3 (0.6)	45.2 (0.8)	
Fluctuating	745	5.5 (0.3)	19.9 (2.1)	23.0 (2.2)	20.6 (2.2)	36.5 (2.8)	
Downward	149	1.0 (0.1)	13.3 (4.4)	23.3 (5.1)	25.9 (6.1)	37.5 (6.8)	
Stable disadvantage	728	2.6 (0.2)	24.6 (3.0)	24.8 (2.9)	21.2 (3.3)	29.4 (3.9)	

SEP, socioeconomic position.

a Weighted percentages of imputed data. *P* value was calculated for each imputed data set; the highest one is reported.

Table 2 shows standardized direct, indirect, and total effects of SEP at 3 life stages on number of teeth in older age. For the overall sample (not stratified by cohort), factor loadings for all latent variables were significant and substantially high (all βs > 0.50), indicating that the constructed latent variables successfully summarized the information contained in the observed variables. The goodness-of-fit statistics indicated that the hypothesized SEP model was an excellent fit to the data. Adult and older age SEP had direct effects on number of teeth, suggesting that higher SEP in these 2 sensitive periods was associated with having more teeth in older age (adulthood, direct β = 0.182, *P* < 0.001; older age, direct β = 0.093, *P* = 0.005). However, the childhood SEP direct path estimate was negative and not significant (direct β = −0.057, *P* = 0.145). Childhood SEP had an indirect effect (indirect β = 0.130, *P* < 0.001) on number of teeth via adult SEP and older age SEP, indicating chain of risks. Adult SEP and older age SEP together accounted for 70.0% of the total effect between SEP across the life course and number of teeth, while older age SEP alone accounted for 24.0% of the total effect of SEP in adulthood on number of teeth.

**Table 2.** Standardized Factor Loadings for Observed Variables and Standardized Coefficients for Total, Direct, and Indirect Effects of SEP in Childhood, Adulthood, and Older Age on Oral Health (*n* = 15,136).

	Standardized Coefficient by Birth Year, β				
	All	1941 or Later	1931 to 1940	1921 to 1930	1920 or Earlier
**Confirmatory factor analysis**					
Childhood SEP by					
Father’s education	0.845^***^	0.832^***^	0.801^***^	0.801^***^	0.726^***^
Father’s main occupation	0.806^***^	0.800^***^	0.831^***^	0.898^***^	0.964^***^
Adult SEP by					
Education	0.897^***^	0.860^***^	0.851^***^	0.831^***^	0.883^***^
Main occupation	0.828^***^	0.875^***^	0.901^***^	0.979^***^	0.993^***^
Older age SEP by					
Self-rated economic status	0.464^***^	0.441^***^	0.542^***^	0.562^***^	0.584^***^
Household income	0.807^***^	0.787^***^	0.823^***^	0.789^***^	0.762^***^
**Path analysis**					
Direct effects					
*a*: childhood SEP on teeth no.	–0.057	–0.052	–0.114	0.018	0.298
*b*: adult SEP on teeth no.	0.182^***^	0.183^**^	0.338^***^	0.234^*^	–0.309
*c*: older age SEP on teeth no.	0.093^**^	0.100^*^	0.040	–0.055	0.139
*d*: childhood SEP on adult SEP	0.540^***^	0.539^***^	0.565^***^	0.489^***^	0.732^***^
*e*: adult SEP on older age SEP	0.628^***^	0.613^***^	0.678^***^	0.746^***^	0.637^***^
Indirect effects					
*db* + *dec*: childhood SEP → teeth no.	0.130^***^	0.132^***^	0.207^***^	0.094^*^	–0.161
*ec*: adult SEP → teeth no.	0.058^**^	0.061^*^	0.027	–0.041	0.089
Total effects: direct effects + indirect effects					
Childhood SEP on teeth no.	0.073^**^	0.079^*^	0.093^**^	0.112^*^	0.137
Adult SEP on teeth no.	0.241^***^	0.244^***^	0.366^***^	0.193^*^	–0.220
Older age SEP on teeth no.	0.093^**^	0.100^*^	0.044	–0.055	0.139
Fit statistics					
χ^2^	469.68	667.73			
*df*	29	116			
Root mean square error of approximation	0.032	0.035			
Standardized root mean squared residual	0.039	1.557			
Comparative fit index	0.962	0.971			
Tucker-Lewis index	0.928	0.946			

Demographic characteristics were adjusted and omitted in all models. Analysis of imputed data set.

SEP, socioeconomic position.

* *P* < 0.05. ^**^P < 0.01. ^***^P < 0.001.

The associations between SEP and number of teeth varied among birth cohorts (Table 2). The multiple-group analysis fitted the data well. All factor loadings for the latent SEP constructs in 3 periods were significant across the 4 cohorts. Substantial direct effects of adult SEP on number of teeth in the 3 younger cohorts (those born in 1941 or later, 1931 to 1940, and 1921 to 1930) suggested that adulthood was a sensitive period for tooth retention. A direct effect of older age SEP on number of teeth was observed only in the youngest cohort, demonstrating that older age was a sensitive period for those born in 1941 or later. Also, indirect effects of childhood SEP were found in the 3 younger cohorts, pointing toward chains of risks.

**Figure 2.**
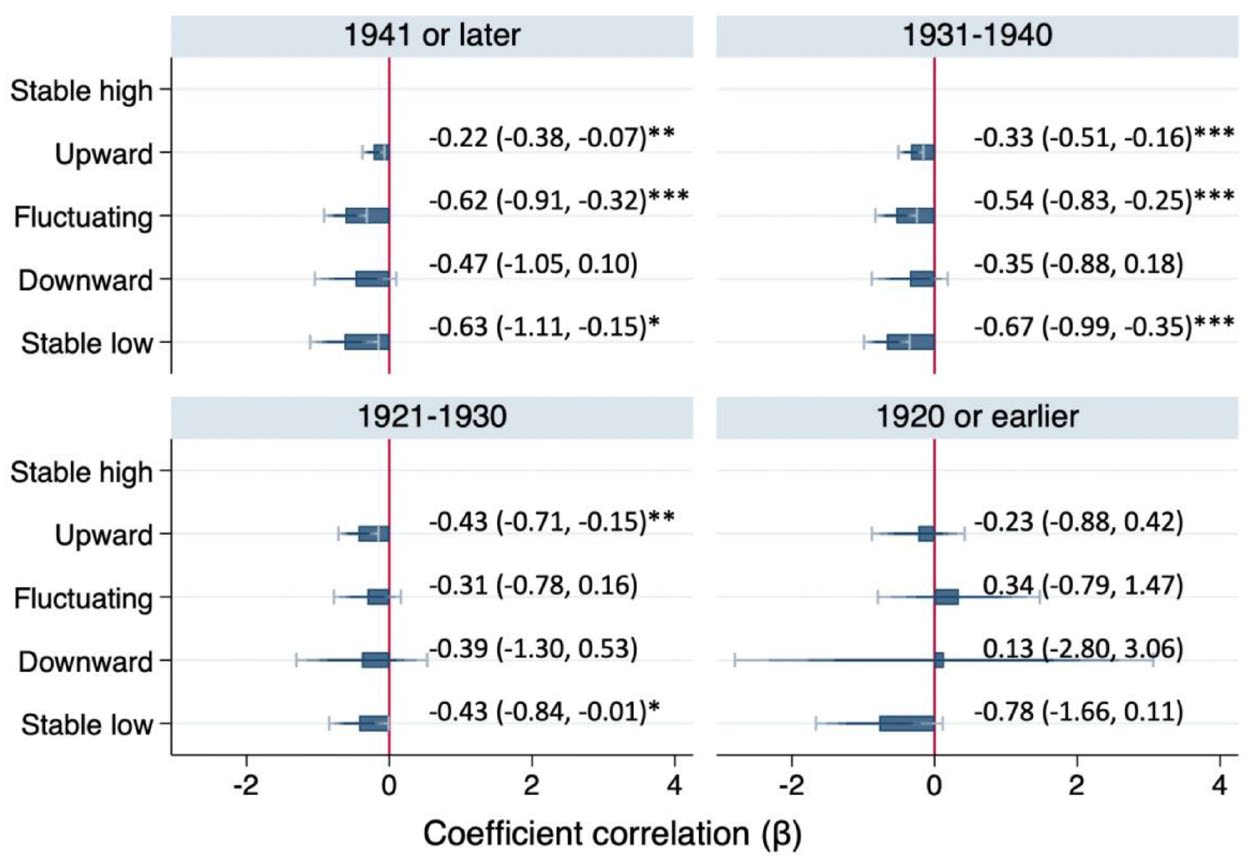
Adjusted associations between social mobility and number of teeth by birth cohort (*n* = 15,136). Analysis of imputed data set. Proportional odds models, all *P* values >0.01 in Brant tests. Demographic characteristics were adjusted. Coefficients (βs) are reported in the social mobility model because 1 estimate of the oldest cohort is relatively large as compared with other estimates. The reference group is stable high socioeconomic position. Data in parentheses indicate 95% CI. *P < 0.05. **P < 0.01. ***P < 0.001.

Due to violation of the proportional odds assumption, a partial proportional odds model was used to obtain estimates for each dichotomization of the outcome. Table 3 reveals that the odds of having functional dentition for those who experienced upward social mobility, fluctuating social mobility, and stable low SEP between childhood and older age were 17% (OR, 0.83; 95% CI, 0.72 to 0.95), 42% (OR, 0.58; 95% CI, 0.44 to 0.76), and 48% (OR, 0.52; 95% CI, 0.35 to 0.78) lower than for those who experienced a stable high SEP, respectively. In PO analyses stratified by birth cohort, graded patterns were observed only in the 3 younger cohorts (Figure 2).

**Table 3.** Association Between Social Mobility Trajectories and Number of Teeth (*n* = 15,136).

	Social Mobility Model, Odds Ratio (95% CI)	
	PO Model^a^	Partial PO Model^b^
Social mobility trajectory: childhood–adulthood–older age		
Stable high	1.00 (Reference)	
Upward	0.78 (0.69 to 0.88)^***^	
Fluctuating	0.56 (0.45 to 0.71)^***^	
Downward	0.65 (0.41 to 1.01)	
Stable low	0.55 (0.41 to 0.74)^***^	
(Edentate = 0) vs. (1 to 9 + 10 to 19 + ≥20 teeth = 1)		
Stable high		1.00 (Reference)
Upward		0.75 (0.63 to 0.89)^**^
Fluctuating		0.58 (0.42 to 0.80)^**^
Downward		0.91 (0.41 to 2.00)
Stable low		0.60 (0.40 to 0.89)^*^
(Edentate + 1 to 9 teeth = 0) vs. (10 to 19 + ≥20 teeth = 1)		
Stable high		1.00 (Reference)
Upward		0.76 (0.65 to 0.87)^***^
Fluctuating		0.55 (0.43 to 0.72)^***^
Downward		0.69 (0.40 to 1.19)
Stable low		0.56 (0.40 to 0.78)^**^
(Edentate + 1 to 9 + 10 to 19 teeth = 0) vs. (≥20 teeth = 1)		
Stable high		1.00 (Reference)
Upward		0.83 (0.72 to 0.95)^**^
Fluctuating		0.58 (0.44 to 0.76)^***^
Downward		0.56 (0.30 to 1.04)
Stable low		0.52 (0.35 to 0.78)^**^

Estimates of covariates were omitted. Weighted percentages of imputed data.

PO, proportional odds.

a Results of the PO model, holding the parallel-lines assumption.

b Results of the partial PO model, which relaxes the assumption of PO for social mobility trajectories (Brant test, χ^2^ =14.77, *P* < 0.01) and assumes PO for covariates (age, sex, region, and rural/urban residence).

* *P* < 0.05. ^**^P < 0.01. ^***^P < 0.001.

Sensitivity analyses for the accumulation of risk model suggested a clear negative social gradient (Appendix 4). Overall, a higher lifetime exposure to disadvantaged SEP was associated with reporting fewer teeth in old age. When stratified by birth cohorts, a greater life course exposure to disadvantaged SEP was associated with having fewer teeth in older age among only the 3 younger cohorts.

## Discussion

Using the Chinese older adult population to investigate associations between life course SEP and number of teeth, this study showed that the number of remaining teeth is subject to marked social inequalities. Our findings document the simultaneous applicability of life course models and a widening of oral health inequalities in China across generations.

Adulthood and older age were sensitive periods for number of teeth in later life, during which Chinese older adults were particularly sensitive to socioeconomic disadvantage (Green and Popham 2017). The particularly strong influence of later SEP on oral health might be due to its immediate and stronger influence on health behaviors and access to care, making it more impactful in shaping health outcomes in older age (Ben-Shlomo and Kuh 2002). We also observed no direct effect but a strong indirect effect of childhood SEP on number of teeth in later life, in line with a study on edentulousness among Chinese adults aged ≥45 y (Zhang et al. 2023) and a relevant Swedish longitudinal study (Celeste et al. 2020). These findings support the accumulation of risk model, suggesting that the effect of childhood SEP on oral health is long-lasting and cumulative, while reflecting social mobility that is intergenerational (childhood to adulthood) and intraindividual (adulthood to older age), providing support for the social mobility model. However, other studies conducted in HICs found that childhood is a sensitive period for oral health (Bernabé et al. 2011; Han and Khang 2017; Lee 2019; Celeste et al. 2020). The different findings in HICs and China might be explained by China’s unique social and economic historical transformation. All participants in the present study were born before 1954 and spent their childhood before the economic reform in 1978, when China was mostly a preindustrial society with low literacy rates and a predominantly rural population. Access to dental services was very limited, as China had one of the lowest dentist-to-population ratios in the world (Schattner and Tsao 1981). Government funding and policies to improve oral health were lacking, with the focus being primarily on communicable diseases (Zhou et al. 2013). However, those from higher SEP households would have been more likely to access expensive sugary products, tobacco, and alcohol in their youth (Chen et al. 2010). In this context, the socioeconomic advantage that granted access to dental services may have been offset by the higher sugar intake and other risk factors associated with more affluent lifestyles. These mutual protective and harmful effects might explain why childhood SEP had no direct effects on number of teeth in our sample.

We observed a social gradient in relation to the accumulation of socioeconomic disadvantage and number of teeth, corresponding to the accumulative risk model and corroborating studies conducted in HICs (Bernabé et al. 2011; Han and Khang 2017; Ramsay et al. 2018; Lee 2019; Celeste et al. 2020). Consistent with previous work testing the social mobility model (Pearce et al. 2009; Astrom et al. 2015; Han and Khang 2017; Ramsay et al. 2018; Lee 2019; Celeste et al. 2022), having a stable advantaged SEP across the life course was most beneficial for retaining more teeth in later life. However, differences between the stable advantaged and upwardly mobile groups were larger here than in other studies (Han and Khang 2017; Ramsay et al. 2018; Lee 2019). This might be explained by the strong effects of later SEP observed in our study, implying that upward mobility helped to compensate for early-life disadvantage. Meanwhile, the larger effect of fluctuating mobility, which includes upward and downward mobility, could be explained by the direct effects of later SEP, as the effect of childhood SEP passes through a chain of SEP in adulthood and older age.

The multiple-group analysis documented cohort effects, with adulthood identified as a sensitive period for number of teeth among the 3 younger cohorts and older age as a sensitive period among the youngest group (born in 1941 or later). The lack of associations between SEP across the life course and number of teeth among the oldest cohort may reflect the low priority of dental care and high prevalence of periodontal diseases among all SEP groups in China before 1980, when this cohort entered old age (Schattner and Tsao 1981). Throughout older adults’ lives in 20th-century China, the participants and their parents were exposed to dramatic social and economic changes, including wars, famine, revolutions, education reforms, industrialization, and a massive increase in incomes. Therefore, the findings might reflect the remarkable social and economic developments in China over the past 4 decades that have contributed to health improvement, while social inequalities in health have also increased (Kendig et al. 2017). Additionally, effects of cumulative disadvantage and social mobility were found among the 3 younger cohorts, due to the higher social inequalities among them.

A particular strength of this study is the examination of 4 life course models in relation to oral health among older adults in China. Identifying sensitive periods, exploring the accumulation of risks and pathways throughout the life course, and understanding the pattern of oral health inequalities can help guide interventions conducive to healthy longevity and oral health improvement in China. Additionally, the CLHLS has a high response rate and a large sample of the oldest-old, while survey weights and multiple imputation reduced bias and improved generalizability. To assess older age SEP, latent variables were constructed to capture subjective socioeconomic status and objective annual household income. Subjective measures are considered to reflect not only people’s current objective social circumstances but also their past SEP (Singh-Manoux et al. 2003) and are widely used as SEP indicators in studies conducted in China (Wong et al. 2008).

Our study has some limitations. First, childhood and adult SEP was captured retrospectively and therefore subject to recall bias. However, studies examining the validity of long-term recall data have demonstrated that individuals can report education, occupation, and childhood adversities with reliability and accuracy (Hout and Hastings 2016). Some important SEP measures (e.g., household income in childhood and adulthood) were not available and could not be included in the analysis, while education and social class had to be dichotomized. To partly address these concerns, latent variables were constructed by including 2 SEP variables at each life stage. Second, the CLHLS does not collect information on oral health at earlier life stages, and it is possible that some tooth loss might precede adult and older age SEP. Finally, downward social mobility was rare (*n* = 86 for the overall sample, *n* = 5 for the oldest cohort), limiting statistical power to detect differences with this group.

Our findings have implications for potential interventions to promote good oral health. Given the pivotal role of adult SEP, interventions to reduce social inequalities—such as increasing access to education, reducing unemployment, and ensuring good physical and psychosocial working conditions for manual and nonmanual workers—can be relevant in this context (Kaikkonen et al. 2009). Meanwhile, social and welfare support to families with children, such as the provision of parental education and universal high-quality nursery/school education, might break the transmission and accumulation of social disadvantage, improve health, and reduce social inequalities (Raudenbush and Eschmann 2015). Oral health inequalities have widened across generations in China, underscoring the urgency for appropriate interventions.

In conclusion, this study provides new important evidence for the influence of SEP across different life stages on oral health among the older Chinese population, also highlighting generational differences. Future research should examine the mechanisms through which oral health inequalities in China are created and develop strategies and interventions to effectively address them.

## Author Contributions

J. Hong, contributed to conception, design, data acquisition and interpretation, performed all statistical analysis, drafted and critically revised the manuscript; R.G Watt, G. Tsakos, A. Heilmann, contributed to conception, design, critically revised the manuscript. All authors have their final approval and agree to be accountable for all aspects of work.

## Supplemental Material

sj-docx-1-jct-10.1177_23800844241297533 – Supplemental material for Socioeconomic Position and Oral Health in Chinese Older Adults: A Life Course Approach Supplemental material, sj-docx-1-jct-10.1177_23800844241297533 for Socioeconomic Position and Oral Health in Chinese Older Adults: A Life Course Approach by J. Hong, R.G. Watt, G. Tsakos and A. Heilmann in JDR Clinical & Translational Research

## Notes

1. Average exchange rate: US dollar to China renminbi in 2017 was 6.76.

2. Childhood disadvantaged SEP score: 1 (SEP disadvantage/low SEP in childhood) if father’s educational attainment was no schooling AND father’s occupation in childhood was manual or unemployed; 0 (no SEP disadvantage/high SEP in childhood) if father had formal education OR father’s occupation was nonmanual OR both. Adulthood disadvantaged SEP score: 1 (SEP disadvantage/low SEP in adulthood) if participant’s educational attainment was no schooling AND participant’s occupation was manual or unemployed; 0 (no SEP disadvantage/high SEP in adulthood) if participant had formal education OR own occupation was nonmanual OR both. Older age disadvantaged SEP score: 1 (SEP disadvantage/low SEP in older age) if self-rated economic status was poor AND low annual household income; 0 (no SEP disadvantage/high SEP in older age) if self-rated economic status was average or good OR annual household income was medium or high OR both.
