# Supplementary material for: Socioeconomic Position and Oral Health in Chinese Older Adults: A Life Course Approach
Source: JDR Clin Trans Res. 2024 Dec 9;10(2):169–79. doi: 10.1177/23800844241297533 (PMC11894898; doi:10.1177/23800844241297533)
Supplement: sj-docx-1-jct-10.1177_23800844241297533 – Supplemental material for Socioeconomic Position and Oral Health in Chinese Older Adults: A Life Course Approach [file sj-docx-1-jct-10.1177_23800844241297533.docx]

# Supplementary appendix

**Socioeconomic position and oral health in Chinese older adults: a life course approach**

Jialan Hong^1, 2, 3 *^, PhD; Richard G Watt^1^, PhD; Georgios Tsakos^1^, PhD; Anja Heilmann^1^, PhD

^1^ Department of Epidemiology and Public Health, University College London, London WC1E 6BT, UK

^2^ National Institute for Health Research Applied Research Collaboration West (NIHR ARC West), University Hospitals Bristol and Weston NHS Foundation Trust, Bristol, UK

^3^ Population Health Sciences, Bristol Medical School, University of Bristol, Bristol, UK

*corresponding author, email: jialan.hong@bristol.ac.uk

**Table of Contents:**

| Appendix 1. Study population and sampling procedure | 2 |
| --- | --- |
| Appendix 2. Justification of using categorical number of teeth as the outcome variable | 3 |
| Appendix 3. Multiple imputation diagnostics and multicollinearity diagnostics  Appendix 4. Sensitivity analysis for the accumulative risks model | 4  9 |
| Appendix References | 11 |
|  |  |

**List of Figures:**

| **Appendix Figure 1 The graphic comparison of observed and predicted probability of each tooth count (weighted result).**  **Appendix Figure 2 Father’s education imputation diagnostic plot**  **Appendix Figure 3 Father’s occupation imputation diagnostic plot**  **Appendix Figure 4 Education imputation diagnostic plot**  **Appendix Figure 5 Occupation imputation diagnostic plot**  **Appendix Figure 6 Household income imputation diagnostic plot**  **Appendix Figure 7 Self-rated economic status imputation diagnostic plot**  **Appendix Figure 8 Adjusted associations between cumulative disadvantage and number of teeth by birth cohort (n=15,136)** | 3  4  5  5  6  6  7  10 |
| --- | --- |

**List of Tables:**

| **Appendix Table 1 Estimated Individual Effects of SEP indicators on number of teeth (Estimate, SE), Adjusted for age, sex, residence area, and region** | 7 |
| --- | --- |
| **Appendix Table 2 Correlation matrix between measures** | 8 |
| **Appendix Table 3 Multicollinearity Diagnostics**  ***Appendix Table 4 Association between cumulative disadvantage and number of teeth (n=15,136)*** | 8  9 |

##### Study population and sampling procedure

Since the inception of the CLHLS in 1998, eight survey waves have been conducted in the period between 1998 and 2018. In the CLHLS, participants aged 65 and over were selected from 50% of counties and cities (n=631) in 23 of China’s 31 provinces.^1^ Initially, 22 provinces (Liaoning, Jilin, Heilongjiang, Hebei, Beijing, Tianjin, Shanxi, Shaanxi, Shanghai, Jiangsu, Zhejiang, Anhui, Fujian, Jiangxi, Shandong, Henan, Hubei, Hunan, Guangdong, Guangxi, Sichuan and Chongqing) were involved in the CLHLS. Hainan was included in the 5th, 6th, and 7th waves as the 23rd province to perform the more in-depth study for longevity areas. The population in these survey areas was 985 million in 1990, constituting approximately 85% of the total population in China at that time. Some areas were not included in the study because of proven significant misreporting of age in population censuses.^2^ The sampling design of CLHLS adopted a multi-stage disproportionate and targeted random sampling method. All centenarians who voluntarily participated and offered informed consent in the sampled sites were eligible to participate, and were randomly assigned a code.^1^ Each participating centenarian was then matched with one octogenarian and one nonagenarian of pre-specified age and sex in nearby villages or districts. “Nearby” is loosely defined, it could be in the same village or on the same street, if available, or in the same town or in the same sampled county or city. This sampling method ensures comparable numbers of randomly selected male and female octogenarians and nonagenarians at each age from 80-99. In the 2002 and later waves, three nearby elders aged 65–79 of predefined age and sex were interviewed in conjunction with every two centenarians.^1^ In the 2008 wave, one nearby un-related middle age control participant aged 40–64 for each of centenarian were recruited. Sub-samples were added in 2002 and 2005 waves - elderly interviewees’ adult children aged 35–65. Participants aged 90 and above were over-sampled to achieve a sufficiently large number of the oldest old (centenarians: octogenarian: nonagenarian: elderly aged 65-79 = 1: 1: 1: 1.5). The dates of death of deceased participants were obtained from police registry and death certificates, or next of kin and local residential committees if death certificates were not available.^1^ In order to ensure that the total sample size remained roughly the same and that the samples were comparable from survey to survey, elderly people who had died or failed to respond to follow-up surveys were replaced by participants nearby from the same region, and of the same sex and roughly the same age. The weights for the over-sampled extremely old persons (e.g. 90+) are less than 1.0, and weights for under-sampled elders (e.g. age 80-85) are greater than 1.0.

##### Justification of using categorical number of teeth as the outcome variable

The number of natural teeth is a measure of accumulation of oral disease and damage over the lifetime.^3^ Previous validation studies of oral health measures supported the validity of self-reported number of natural teeth reflected the clinically-examined number of teeth.^4,5^

In the CLHLS, participants were asked to count the number of natural teeth present under the assistance of the medical personnel. The number of natural teeth of participants ranged from 0 to 32. This variable is available across all eight waves of the CLHLS. The distribution of number of teeth is markedly skewed, with 15·3% of the participant having zero teeth and the probability of having each tooth count from 1 to 32 was less than 10% (weighted).

Therefore, the zero-inflation assumption of this count variable was tested by using participant’s education level as a predictor of number of teeth. Appendix Figure 1 showed that the predicted zero-inflated negative binomial (ZINB) distribution fits the observed probability of the number of teeth better than Poisson and negative binomial distribution. However, there was heterogeneity between observed distribution and ZINB distribution. Therefore, the discretization of the count variable was performed to achieve better model fit and capture the non-linear pattern. Although it has been discussed that discretization might result in loss of power, information loss can be substantially reduced by selecting the cut-off points adaptively and keeping the interpretability of the categorisation.^6,7^ Accordingly, the number of teeth were grouped into 4 ordered groups to achieve better model fit and capture the non-linear pattern of number of teeth: edentulousness (0 teeth), 1-9 teeth, 10-19 teeth, functional dentition (20-32 teeth). We further examined the proportional-odds assumption. The p-value of the likelihood-ratio test (assumption: proportional odds model nested in non-proportional odds model) was <0.001, therefore, we accepted the hypothesis that relaxing the proportional-odds for explanatory variable does not improve the model.

**
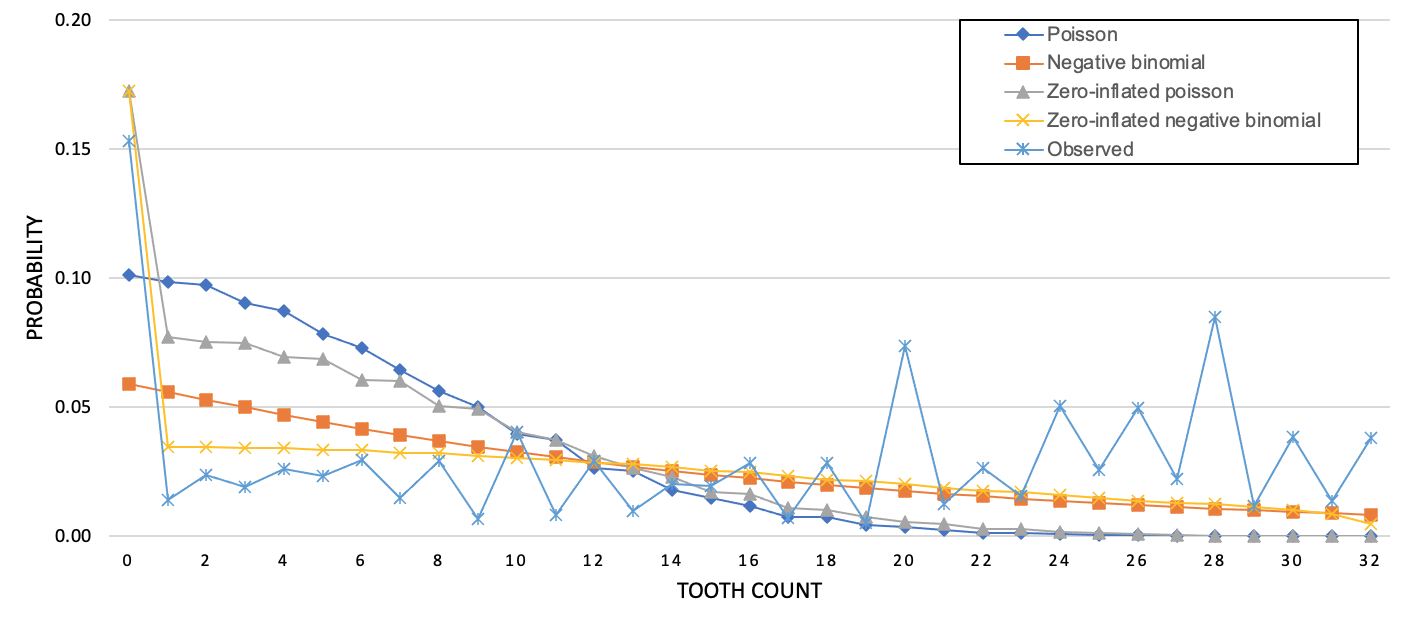
**

**Appendix Figure 1 The graphic comparison of observed and predicted probability of each tooth count (weighted result).**

To choose the model that fit the number of teeth in the CLHLS dataset, comparison between regular poisson regression model (PRM), negative binomial regression model (NBRM), zero-inflated poisson (ZIP) and zero-inflated negative binomial model (ZINB). Result showing that ZINB is the best model because the residual from all the tested models showed a close to zero residue. Small residuals are indicative of good-fitting models, so the models with lines closest to zero should be considered for our data. Zero-inflated negative binomial model fits better than zip model at any count points.

##### Multiple imputation diagnostics and multicollinearity diagnostics

**Multiple imputation diagnostics**

Figures show the diagnostic plots for the predicted distributions of father’s education, father’s occupation, individual’s education, individual’s occupation, self-rated economic status, and household income produced during thirty-five imputations. The plots showed that the values vary randomly.

Appendix Figure 2 Father’s education imputation diagnostic plot

Appendix Figure 3 Father’s occupation imputation diagnostic plot

Appendix Figure 4 Individual’s education imputation diagnostic plot

Appendix Figure 5 Individual’s occupation imputation diagnostic plot

Appendix Figure 6 Household income imputation diagnostic plot

Appendix Figure 7 Self-rated economic status imputation diagnostic plot

Appendix Table 1 shows coefficient estimates and standard errors from complete case analysis and multiple imputation are similar.

***Appendix Table 1 Estimated Individual Effects of SEP indicators on number of teeth (Estimate, SE), Adjusted for age, sex, residence area, and region***

| SEP indicator | Complete case analysis | Multiple imputation analysis |
| --- | --- | --- |
| Father's education (n=14,228) |  |  |
| 0 years |  |  |
| ≥ 1 year | 0.18 (0.059) | 0.19 (0.059) |
| Father's occupation in childhood (n=14,804) | |  |
| Manual or unemployed |  |  |
| Non-mannual | 0.05 (0.118) | 0.05 (0.117) |
| Own education (n=14,923) |  |  |
| 0 years |  |  |
| 1-6 years | 0.13 (0.060) | 0.13 (0.060) |
| ≥ 7 years | 0.60 (0.072) | 0.59 (0.071) |
| Main occupation (n=14,904) |  |  |
| Manual or unemployed |  |  |
| Non-mannual | 0.55 (0.082) | 0.55 (0.082) |
| Self-rated economic status (n=14,966) |  |  |
| Poor |  |  |
| Average | 0.24 (0.077) | 0.24 (0.076) |
| Good | 0.48 (0.091) | 0.48 (0.090) |
| Annual household income (n=13,825) |  |  |
| Low, <¥10,000 |  |  |
| Medium, ¥10,000-49,999 | 0.09 (0.061) | 0.10 (0.060) |
| High ≥¥50,000 | 0.43 (0.064) | 0.43 (0.064) |
| Social mobility trajectories (n=12,761) |  |  |
| Stable high |  |  |
| Upward | -0.25 (0.066) | -0.25 (0.063) |
| Fluctuating | -0.57 (0.120) | -0.58 (0.117) |
| Downward | -0.53 (0.239) | -0.43 (0.228) |
| Stable low | -0.57 (0.156) | -0.59 (0.151) |

**Multicollinearity diagnostics**

**Step 1:** To explore the correlation matrix between each SEP measures and display all pairwise correlation coefficients, we implemented the STATA PWCORR with survey weight using twenty multiple imputed datasets. The correlation coefficients ranged from 0.07 to 0.45. Upon review of the correlation matrix, no variables with a particularly high correlation (coefficient ≥ 0.8) were observed.

**Appendix Table 2 Correlation matrix between measures**

|  | | 1 | | 2 | | 3 | | 4 | | 5 | | 6 | |
| --- | --- | --- | --- | --- | --- | --- | --- | --- | --- | --- | --- | --- | --- |
| *1.     Father's education* | | 1.00 | |  | |  | |  | |  | |  | |
| *2.     Father's occupation in childhood* | | 0.32* | | 1.00 | |  | |  | |  | |  | |
| *3.     Own education* | | 0.36* | | 0.21* | | 1.00 | |  | |  | |  | |
| *4. Main occupation before age 60* | | 0.22* | | 0.22* | | **0.45*** | | 1.00 | |  | |  | |
| *5.     Self-rated economic status* | | 0.10* | | **0.07*** | | 0.19* | | 0.20* | | 1.00 | |  | |
| *6.     Annual household income* | | 0.17* | | 0.11* | | 0.25* | | 0.26* | | 0.32* | | 1.00 | |

*Analysis of twenty imputed datasets*

** indicates p<0.050*

**Step 2:** To examine multicollinearity through the Variance Inflation Factor (VIF) and Tolerance, the STATA COLLIN command was used. There were no values fall below 0·1 for “Tolerance”, and no values above 10 for “VIF”. Therefore, a lack of multicollinearity of these variables of the dataset were confirmed by the multicollinearity diagnostics.

**Appendix Table 3 Multicollinearity Diagnostics**

| **Multicollinearity Diagnostics** | | | | | | |
| --- | --- | --- | --- | --- | --- | --- |
| Variable | Eigen Value | Condition Index | VIF | Sort VIF | Tolerance | R- Squared |
| *Father's education* | 2.1698 | 1.0000 | 1.24 | 1.12 | 0.8035 | 0.1965 |
| *Father's occupation in childhood* | 1.1026 | 1.4028 | 1.13 | 1.06 | 0.8838 | 0.1162 |
| *Own education* | 0.8384 | 1.6087 | 1.41 | 1.19 | 0.7076 | 0.2924 |
| *Main occupation before age 60* | 0.7139 | 1.7434 | 1.32 | 1.15 | 0.7596 | 0.2404 |
| *Self-rated economic status* | 0.6717 | 1.7973 | 1.13 | 1.07 | 0.8814 | 0.1186 |
| *Annual household income* | 0.5036 | 2.0756 | 1.20 | 1.09 | 0.8365 | 0.1635 |

##### Sensitivity analysis for the accumulative risks model

**Appendix Table 4 Association between cumulative disadvantage and number of teeth (n=15,136)**

|  | Accumulative risks model –  Odds Ratio (95% Confidence Interval) | |
| --- | --- | --- |
|  | PO model^i^ | PPO model^ii^ |
| **Periods of experienced disadvantaged SEP** | | |
| 0 periods | 1.00 (reference) |  |
| 1 period | 0.81 (0.71-0.92)** |  |
| 2 periods | 0.67 (0.59-0.78)*** |  |
| 3 periods | 0.53 (0.40-0.72)*** |  |
|  |  |  |
| (Edentate = 0) v.s. (1-9 teeth + 10-19 teeth + ≥20 teeth = 1) | | |
| 0 periods |  | 1.00 (reference) |
| 1 period |  | 0.71 (0.58-0.85)*** |
| 2 periods |  | 0.75 (0.62-0.91)** |
| 3 periods |  | 0.59 (0.40-0.88)** |
|  |  |  |
| (Edentate + 1-9 teeth = 0) v.s. (10-19 teeth + ≥20 teeth = 1) | | |
| 0 periods |  | 1.00 (reference) |
| 1 period |  | 0.79 (0.67-0.92)** |
| 2 periods |  | 0.65 (0.55-0.76)*** |
| 3 periods |  | 0.55 (0.40-0.77)*** |
|  |  |  |
| (Edentate + 1-9 teeth + 10-19 teeth = 0) v.s. (≥20 teeth = 1) | | |
| 0 periods |  | 1.00 (reference) |
| 1 period |  | 0.86 (0.74-0.99)* |
| 2 periods |  | 0.69 (0.59-0.81)*** |
| 3 periods |  | 0.52 (0.35-0.77)** |
|  |  |  |

Estimates of covariates were omitted. Weighted percentages of imputed data. ***p<0.001; **p<0.01; *p<0.05.

^i^ results of the proportional odds model (PO), holding the parallel-lines assumption.

^ii^ results of the partial proportional odds model (PPO), which relax the assumption of proportional odds for cumulative disadvantaged SEP (Brant’s test: χ^2^_cumulative disadvantage_=22.38) and assume proportional odds for covariates (age, sex, region, and rural/urban residence).

Appendix Figure 8 Adjusted associations between cumulative disadvantage and number of teeth by birth cohort (n=15,136)

***p<0.001; **p<0.01; *p<0.05. Analysis of imputed dataset. PO Models, all p-values > 0.01 in Brant’s tests. Demographic characteristics were adjusted. Cumulative risk model. Odds ratios (ORs) are reported in the cumulative risk model. The reference group is disadvantaged SEP in 0 periods.

**Appendix References**

1. Zeng Y, Poston DL, Vlosky DA, Gu D. Healthy Longevity in China: Demographic, Socioeconomic, and Psychological Dimensions: Springer Science & Business Media; 2008.

2. Zheng Z. Twenty years’ follow-up on elder people’s health and quality of life. *China Population and Development Studies* 2020; **3**(4): 297-309.

3. Shen J, Wildman J, Steele J. Measuring and decomposing oral health inequalities in an UK population. *Community Dentistry and Oral Epidemiology* 2013; **41**(6): 481-9.

4. Douglass CW, Berlin J, Tennstedt S. The validity of self‐reported oral health status in the elderly. *J Public Health Dent* 1991; **51**(4): 220-2.

5. Pitiphat W, Garcia RI, Douglass CW, Joshipura KJ. Validation of self‐reported oral health measures. *J Public Health Dent* 2002; **62**(2): 122-8.

6. O'Brien SM. Cutpoint selection for categorizing a continuous predictor. *Biometrics* 2004; **60**(2): 504-9.

7. Gelman A, Park DK. Splitting a predictor at the upper quarter or third and the lower quarter or third. *The American Statistician* 2009; **63**(1): 1-8.
